# Sequential Therapy of Nadroparin and Rivaroxaban in the Initial Treatment of Patients With Acute Pulmonary Embolism

**DOI:** 10.3389/fphar.2022.810455

**Published:** 2022-03-24

**Authors:** Wei Xiong, Yunfeng Zhao, Song Liu, He Du, Yanmin Wang, Wenjie Li, Xuejun Guo

**Affiliations:** ^1^ Department of Pulmonary and Critical Care Medicine, Xinhua Hospital, Shanghai Jiaotong University School of Medicine, Shanghai, China; ^2^ Department of Pulmonary and Critical Care Medicine, Punan Hospital, Pudong New District, Shanghai, China; ^3^ Department of Medical Oncology, Shanghai Pulmonary Hospital, Tongji University School of Medicine, Shanghai, China; ^4^ Department of Emergency, Xinhua Hospital, Shanghai Jiaotong University School of Medicine, Shanghai, China

**Keywords:** acute pulmonary embolism, LMWH, nadroparin, DOAC, rivaroxaban, initial treatment, sequential therapy

## Abstract

**Background:** Sequential low molecular weight heparin (LMWH) plus warfarin, LMWH plus edoxaban, and LMWH plus dabigatran regimens have already shown efficacy and safety in the treatment of acute pulmonary embolism (PE). The efficacy and safety of sequential LMWH plus rivaroxaban regimen in the treatment of acute PE have been understudied.

**Methods:** A retrospective study was performed to explore the efficacy and safety of sequential therapy regimens of subcutaneous LMWH (nadroparin 86 IU/kg every 12 h for a week) followed by oral rivaroxaban (20 mg once daily for 3 months) for the management of patients with established acute PE without hemodynamic instability, compared with those of nadroparin plus dabigatran and nadroparin plus warfarin.

**Results:** The number of patients with total resolution of PE were 238 (80.1%), 220 (78.0%), and 166 (62.6%), in the nadroparin + rivaroxaban, nadroparin + dabigatra, and nadroparin + warfarin groups, respectively. (*p* = 0.001) The prevalence of DVT at the 3-month follow-up visit was 18 (6.1%), 14 (5.0%), and 11 (4.2%), in the aforementioned three groups, respectively. (*p* = 0.559) The NT-proBNP level (pg/ml) at the 3-month follow-up visit was 122.5 (97.4–158.9), 131.7 (102.2–166.3), and 357.8 (275.4–433.2) in the three groups, respectively. (*p* = 0.001) The D-dimer level (ng/ml) at the 3-month follow-up visit was 387.3 (310.9–465.2), 432.5 (382.4–489.6), and 854.0 (721.5–993.7) in the three groups, respectively (*p* < 0.001). The number of patients with major bleeding events was 3(0.9%), 6(1.8%), and 18 (5.5%) in the three groups, respectively (*p* < 0.001).

**Conclusion:** The regimen of sequential subcutaneous nadroparin at body-weight adjusted dose for a week followed by oral rivaroxaban at a dose of 20 mg once daily for 3 months is effective and safe in the initial treatment of patients with acute pulmonary embolism.

## Introduction

Venous thromboembolism (VTE) is broadly defined as pulmonary embolism (PE), deep venous thrombosis (DVT), superficial vein thrombosis (SVT), and/or splanchnic vein thrombosis (SPVT), (Streiff et al., 2018), whereas narrowly defined as PE and/or DVT (Konstantinides et al., 2019; Konstantinides et al., 2020). Epidemiological statistics demonstrated that annual incidence rates range from 39 to 115 per 100,000 population for PE, whereas DVT incidence rates range from 53 to 162 per 100,000 population. Acute PE is the third most frequent acute cardiovascular syndrome behind myocardial infarction and stroke globally (Konstantinides et al., 2019; Konstantinides et al., 2020).

Systemic thrombolysis is usually used in acute PE patients with hemodynamic instability, whereas anticoagulation is applicable to those without hemodynamic instability (Konstantinides et al., 2019; Konstantinides et al., 2020). Anticoagulation options recommended for the management of PE include monotherapy regimens that use only one type of agent, and combination therapy regimens use more than one type of agent (Streiff et al., 2018). Monotherapy regimens primarily include low molecular weight heparin (LMWH), which are represented by enoxaparin, dalteparin, fondaparinux, rivaroxaban, apixaban etc.,. Combination therapy regimens primarily comprise sequential LMWH plus vitamin K antagonist (VKA) and LMWH plus direct oral anticoagulants (DOACs) that mainly include edoxaban and dabigatran (Kearon et al., 2016; Streiff et al., 2018; Farge et al., 2019; Konstantinides et al., 2019; Key et al., 2020; Konstantinides et al., 2020).

Sequential LMWH plus warfarin, LMWH plus edoxaban, and LMWH plus dabigatran regimens have already shown efficacy and safety in previous clinical trials (Kearon et al., 2016; Streiff et al., 2018; Farge et al., 2019; Konstantinides et al., 2019; Key et al., 2020; Konstantinides et al., 2020). Due to national conditions of China, most patients with acute PE are hospitalized for a treatment of which subcutaneous LMWH for the initiation phase of therapy is preferred over oral anticoagulation. Meanwhile, factor Xa inhibitor is highly recommended for the treatment of acute PE in recent years (Streiff et al., 2018; Farge et al., 2019; Konstantinides et al., 2019; Key et al., 2020; Konstantinides et al., 2020). Nevertheless, edoxaban had not been covered by health insurance until 2020. Accordingly, sequential subcutaneous LMWH for 5–10 days (initiation phase) followed by oral rivaroxaban 20 mg once daily (treatment phase) has become an alternative regimen for the management of PE patients instead of rivaroxaban monotherapy which is rivaroxaban 15 mg twice daily for 21 days followed by 20 mg once daily in lots of hospitals in China in recent years. Nevertheless, the efficacy and safety of such regimens have not been sufficiently assessed to date. Accordingly, this study was designed to investigate the efficacy and safety of combination therapy regimens of sequential LMWH followed by rivaroxaban in the management of patients with acute PE.

## Methods

### Study Design

A retrospective study was performed to explore the efficacy and safety of sequential therapy regimens of subcutaneous LMWH which was body-weight adjusted nadroparin followed by oral rivaroxaban at a dose of 20 mg once daily in the management of patients with established acute PE, which included symptomatic or incidental PE without hemodynamic instability. The efficacy and safety were compared among the nadroparin + rivaroxaban group in which patients received sequential nadroparin plus rivaroxaban, the nadroparin + dabigatran group in which the patients received sequential nadroparin plus dabigatran, and the nadroparin + warfarin group in which the patients received sequential nadroparin plus warfarin, from the baseline which was defined as the diagnoses of acute PE through the 3-month follow-up. Based on the matching of body weight, anatomical extent of PE (EINSTEIN–PE Investigators et al., 2012), and DVT prevalence, the proportion of number of patients in the aforementioned three groups was determined to be approximately 1:1:1.

The administration of subcutaneous nadroparin was body-weight adjusted at a dose of 86 IU (international units)/kg (kilogram) every 12 h for a week. Rivaroxaban was administered at a dose of 20 mg once daily for 3 months following LMWH. Dabigatran was administered at a dose of 150 mg twice daily for 3 months following LMWH. Warfarin was administered at a dose of 2.5–5 mg once daily concurrently with LMWH for at least 5 days until an international normalized ratio (INR) ≥2.0 was achieved for 24 h. Then, the dose of warfarin was adjusted to maintain an INR of 2.0–3.0 for 3 months.

The primary outcomes were the results of re-examination of PE at the follow-up visit after 3 months of the aforementioned anticoagulant treatment. Re-examination of PE was performed by using computed tomography pulmonary angiography (CTPA) and/or planar ventilation/perfusion (V/Q) scan. Based on the results of reexamination of PE, the patients were dichotomized into the resolution group that was defined as the absence of PE on CTPA and V/Q scan and the nonresolution group that was defined as the remnant presence of PE on CTPA and/or V/Q scan. The resolution rate of PE in each group was compared among the nadroparin + rivaroxaban, nadroparin + dabigatran, and nadroparin + warfarin groups. The resolution rate of PE in each group was defined as the proportion of number of patients whose PE was resolved in the total number of patients of each group at the 3-month follow-up visit. Another primary outcome was the prevalence of DVT on compression ultrasonography (CUS) in each group at the 3-month follow-up visit. The prevalence of DVT was compared among the aforementioned three groups.

The secondary outcomes were the cardiac troponin I level, NT-proBNP level, D-dimer, and probability of pulmonary hypertension (PH) on transthoracic echocardiogram at the 3-month follow-up visit. Probability of PH was dichotomized into PH-likely and PH-unlikely based on the criteria in guidelines (Konstantinides et al., 2019; Konstantinides et al., 2020). The cardiac troponin I level, NT-proBNP level, D-dimer, and the prevalence of PH-likely patients at the 3-month follow-up visit were compared among the nadroparin + rivaroxaban, nadroparin + dabigatran, and nadroparin + warfarin groups. The level change of troponin I, NT-proBNP, and D-dimer from the baseline through the 3-month follow-up visit was also compared among the aforementioned three groups. The absolute values of laboratory parameters at the endpoint were compared with those at the baseline for all three groups.

The third outcomes were the events of progressed fatal PE for which thrombectomy, vena cava filter insertion, or thrombolysis were performed or indicated, the death from fatal PE, and the compelled discontinuation of anticoagulation included reduction or cessation due to adverse events, during the 3-month treatment of acute PE. Fatal PE was defined as PE with hemodynamic instability. Adverse events were defined as bleeding including major bleeding and minor one. Major bleeding was defined as fatal bleeding, and/or symptomatic bleeding in a critical area or organ, and/or a fall in the hemoglobin level of 20 g/L or more or a transfusion of two or more units of whole blood or red cells due to bleeding (Schulman and Kearon, 2005). Bleeding that did not conform to major bleeding was defined as minor bleeding. The third outcome was compared among the nadroparin + rivaroxaban, nadroparin + dabigatran, and nadroparin + warfarin groups.

The principal safety outcomes had major bleeding from the initiation of anticoagulation through the 3-month follow-up visit. Major bleeding rates were compared among the nadroparin + rivaroxaban, nadroparin + dabigatran, and nadroparin + warfarin groups. The major bleeding rate was defined as the percentage of patients with at least one episode of major bleeding in each group. For patients with major bleeding, anticoagulation was stopped and not recovered until the bleeding stopped. A vena cava filter insertion or thrombectomy was performed in case of persistent bleeding. For those patients who returned for a next follow-up visit at 6 months, the shared major risk factors for both VTE recurrence and major bleeding were analyzed.

The primary and secondary outcomes were not compared among the aforementioned three groups for patients with progressed fatal PE for which thrombectomy, vena cava filter insertion, or thrombolysis were performed; those who died of fatal PE resulted from the progression of PE; and those whose anticoagulant was reduced or ceased due to adverse events, during the 3-month treatment of acute PE.

This study was performed by the investigators of Shanghai Xinhua Hospital, Shanghai Pulmonary Hospital, and Shanghai Punan Hospital. All data needed for the study were retrieved from the electronic medical record system (EMRS) of each participating hospital. All authors vouch for the completeness and accuracy of the data. No one who is not an author contributed to the writing of the manuscript. The study protocol was approved by the institutional review board of each participating hospital.

### Study Population

Eligible patients from each participating hospital were retrospectively included into the current study based on the inclusion and exclusion criteria. The inclusion criteria comprised the following: 1) All eligible patients were 18 years old or older; 2) all eligible patients were diagnosed with an established acute PE without hemodynamic instability by using CTPA and/or V/Q scan; 3) all patients underwent anticoagulation from the baseline through the following 3 months. The exclusion criteria comprised the following: 1) Patients who were ineligible to receive the aforementioned standard therapeutic anticoagulation due to contraindications or special situation (Streiff et al., 2018; Farge et al., 2019; Key et al., 2020); 2) patients who had a history of chronic thromboembolic disease (CTED) (Konstantinides et al., 2019; Konstantinides et al., 2020); 3) patients who received anticoagulation prior to the diagnoses of acute PE and/or other anticoagulants besides the aforementioned regimens during the 3-month treatment of acute PE; and 4) patients with a confirmed diagnosis of COVID-19.

### Statistical Analyses

Measurement data were presented as mean ± standard deviation or median with an interquartile range based on if they conformed to normal distribution. The categorical data were presented as percentages. The comparison of measurement data among the nadroparin + rivaroxaban, nadroparin + dabigatran, and nadroparin + warfarin groups was performed by using analysis of variance (ANOVA). The comparison of rates was performed by chi-square test. The comparison of time-to-event cumulative fatal PE events, death due to fatal PE, compelled anticoagulant reduction or cessation due to adverse events, and major bleeding events among the aforementioned three groups was performed by using Kaplan–Meier method. Shared major risk factors for both VTE recurrence and major bleeding were analyzed by using univariate and multivariate logistic regression analyses. Statistical analyses were performed by using SPSS 26. Statistical significance was defined as a *p* value being less than 0.05.

## Results

### Demographics and Characteristics of Patients

**FIGURE 1:**
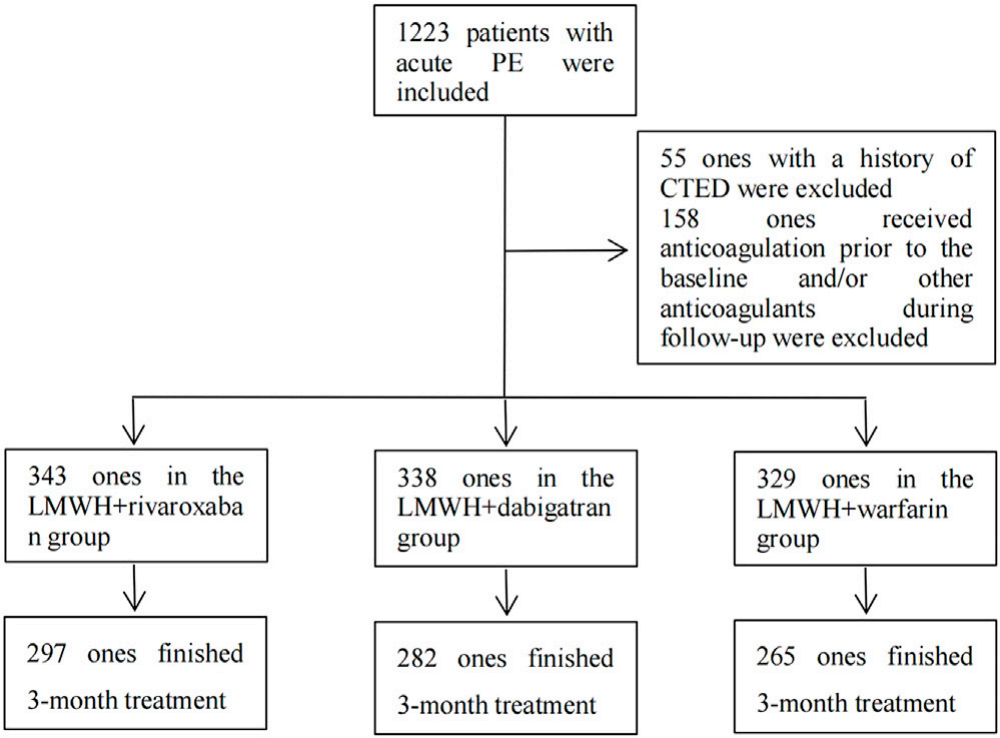
Inclusion, exclusion, and follow-up of patients. Note: PE, pulmonary embolism; CTED, chronic thromboembolic disease; LMWH, low molecular weight heparin.

Based on the inclusion criteria, the data of a total of 1,223 eligible patients from Jan 2010 through Dec 2020 were retrieved from the EMRS of Shanghai Xinhua Hospital, Shanghai Pulmonary Hospital, and Shanghai Punan Hospital. As per the exclusion criteria, 55 patients who had a history of CTED and 158 patients who received other anticoagulants besides the aforementioned regimens during the 3-month treatment of acute PE were excluded. Finally, a total of 1,010 patients with acute PE entered into the final analysis procedure. The number of patients in the nadroparin + rivaroxaban, nadroparin + dabigatran, and nadroparin + warfarin groups was 343, 338, and 329 at the baseline, respectively. The median age of all patients was 65.6 years. The number of male and female patients was 563 and 447, respectively. (Figure 1). The demographic and clinical characteristics of all eligible patients at the baseline are summarized in Table 1.

**TABLE 1:** Demographics and characteristics of patients at the baseline.

Variables	Nadroparin + rivaroxaban (*n* = 343)	Nadroparin + dabigatran (*n* = 338)	Nadroparin + warfarin (*n* = 329)	*p* Value
Mean age-yr	65.6 (53.6–76.4)	67.3 (54.5–79.2)	63.9 (55.2–75.3)	0.781
Male sex-no. (%)	181 (52.8)	205 (60.7)	177 (53.8)	0.077
Weight-kg	62.5 (52.1–77.3)	65.1 (53.5–78.9)	63.8 (51.8–76.4)	0.886
Creatinine clearance ml/min	97.4 (74.2–120.3)	110.5 (87.29–140.4)	78.3 (58.3–101.5)	0.038
Platelet count-×10^9^/L	306.2 (266.7–453.9)	319.4 (257.7–365.8)	278.5 (230.8–324.5)	0.063
Diagnostic methods-no. (%)	—	—	—	0.082
CTPA and V/Q scanning	275 (80.2)	283 (83.7)	245 (74.5)	—
V/Q scanning	68 (19.8)	55 (16.3)	98 (25.5)	—
Risk strata of PE-no. (%)	—	—	—	0.923
Intermediate risk	241 (70.3)	226 (66.9)	235 (71.4)	—
Low risk	102 (29.7)	112 (33.1)	94 (28.6)	—
Anatomical extent of PE-no. (%)	—	—	—	0.958
Limited: ≤25% of vasculature of a single lobe	50 (14.6)	48 (14.2)	49 (14.9)	—
Intermediate	208 (60.6)	206 (60.9)	197 (59.9)	—
Extensive: multiple lobes and >25% of entire pulmonary vasculature	85 (24.8)	84 (24.9)	83 (25.2)	—
Concurrent DVT-no. (%)	86 (25.1)	110 (32.5)	91 (27.7)	0.071
Cause of PE-no. (%)	—	—	—	0.036
Unprovoked	216 (63.0)	201 (59.5)	226 (68.7)	—
Provoked	127 (37.0)	137 (40.5)	103 (31.3)	—
Previous history of VTE-no. (%)	90 (26.2)	111 (32.8)	95 (28.9)	0.382
TNI-ng/mL	0.3 (0.1–0.5)	0.5 (0.2–0.8)	0.2 (0.1–0.3)	0.020
NT-proBNP-pg/mL	1,298.3 (1,093.2–1,520.3)	1,582.4 (1,239.8–1933.4)	1,321.8 (1,022.6–1,643.1)	0.066
D-dimer-ng/mL	2,318.4 (1987.3–2,709.5)	1891.3 (1,443.7–2,308.8)	1,465.9 (1,033.3–1840.9)	0.034
PH-likely-no. (%)	65 (19.0)	78 (23.1)	70 (21.3)	0.631

Note: CTPA, computed tomography pulmonary angiography; V/Q, ventilation/perfusion (lung scintigraphy); PE, pulmonary embolism; DVT, deep venous thrombosis; VTE, venous thromboembolism; TNI, troponin I; NT-proBNP, N-terminal pro B-type natriuretic peptide; PH, pulmonary hypertension.

### Primary Outcomes

After the subtraction of patients with fatal PE, including those who died from fatal PE and those with compelled anticoagulant reduction or cessation due to adverse events during the 3-month follow-up, 297, 282, and 265 patients in the nadroparin + rivaroxaban, nadroparin + dabigatran, and nadroparin + warfarin groups finished the entire 3-month treatment of PE, respectively. (Figure 1). Based on the results of PE imaging reexamination at the 3-month follow-up visit, the number of patients with total resolution of PE was 238 (80.1%), 220 (78.0%), and 166 (62.6%), in the nadroparin + rivaroxaban, nadroparin + dabigatran, and nadroparin + warfarin groups, respectively. (*p* = 0.001) Based on the results of CUS at the 3-month follow-up visit, the prevalence of DVT was 18 (6.1%), 14 (5.0%), and 11 (4.2%), in the aforementioned three groups, respectively (*p* = 0.559) (Table 2).

**TABLE 2:** Primary and secondary outcomes at the 3-month follow-up visit.

Variables	Nadroparin + rivaroxaban (*n* = 297)	Nadroparin + dabigatran (*n* = 282)	Nadroparin + warfarin (*n* = 265)	*p* Value
Primary outcome				
Total resolution-no. (%)	238 (80.1)	220 (78.0)	166 (62.6)	0.001
DVT prevalence-no. (%)	18 (6.1)	14 (5.0)	11 (4.2)	0.559
Secondary outcome				
TNI-ng/mL	0.2 (0.1–0.3)	0.3 (0.1–0.5)	0.1 (0.0–0.2)	0.095
NT-proBNP- pg/mL	122.5 (97.4–158.9)	131.7 (102.2–166.3)	357.8 (275.4–433.2)	0.001
D-dimer-ng/mL	387.3 (310.9–465.2)	432.5 (382.4–489.6)	854.0 (721.5–993.7)	<0.001
Change of TNI-ng/mL	0.1 (0.0–0.2)	0.2 (0.1–0.3)	0.1 (0.0–0.2)	0.453
Change of NT-proBNP-pg/mL	1,175.8 (885.6–1,473.7)	1,450.7 (1,009.5–1857.4)	963.6 (765.7–1,255.7)	0.001
Change of D-dimer-ng/mL	1932.4 (1,256.8–2,674.2)	1,460.4 (1,047.3–1884.7)	611.4 (478.5–833.7)	<0.001
PH-likely-no. (%)	6 (2.0)	8 (2.8)	7 (2.6)	0.285

Note: DVT, deep venous thrombosis; TNI:troponin I; NT-proBNP, N-terminal pro B-type natriuretic peptide; PH, pulmonary hypertension.

### Secondary Outcomes

The cardiac troponin I level (ng/ml) at the 3-month follow-up visit was 0.2 [95% confidence interval (CI) 0.1–0.3], 0.3 (0.1–0.5), and 0.1 (0.0–0.2) in the nadroparin + rivaroxaban (*n* = 297), nadroparin + dabigatran (*n* = 282), and nadroparin + warfarin (*n* = 265) groups, respectively (*p* = 0.095) The NT-proBNP level (pg/ml) at the 3-month follow-up visit was 122.5 (97.4–158.9), 131.7 (102.2–166.3), and 357.8 (275.4–433.2) in the aforementioned three groups, respectively. (*p* = 0.001) The D-dimer level (ng/ml) at the 3-month follow-up visit was 387.3 (310.9–465.2), 432.5 (382.4–489.6), and 854.0 (721.5–993.7) in the aforementioned three groups, respectively (*p* < 0.001). The number of PH-likely patients at the 3-month follow-up visit was 6 (2.0%), 8 (2.8%), and 7 (2.6%) in the aforementioned three groups, respectively. (*p* = 0.285) In addition, the absolute values of troponin I, NT-proBNP, and D-dimer at the endpoint all declined compared with those at the baseline for all three groups (Table 3). The level change of troponin I from the baseline through the 3-month follow-up visit was 0.1 (0.0–0.2), 0.2 (0.1–0.3), and 0.1 (0.0–0.2) in the aforementioned three groups, respectively. (*p* = 0.453) The level change of NT-proBNP from the baseline through the 3-month follow-up visit was 1,175.8 (885.6–1,473.7), 1,450.7 (1,009.5–1857.4), and 963.6 (765.7–1,255.7) in the aforementioned three groups, respectively. (*p* = 0.001) The level change of D-dimer from the baseline through the 3-month follow-up visit was 1932.4 (1,256.8–2,674.2), 1,460.4 (1,047.3–1884.7), and 611.4 (478.5–833.7) in the aforementioned three groups, respectively (*p* < 0.001) (Table 2).

**TABLE 3:** Comparison of laboratory parameters between the baseline and endpoint.

Variables	Nadroparin + rivaroxaban (*n* = 297)			Nadroparin + dabigatran (*n* = 282)			Nadroparin + warfarin (*n* = 265)		
	Baseline	Endpoint	*p* value	Baseline	Endpoint	*p* value	Baseline	Endpoint	*p* value
TNI-ng/ml	0.3 ± 0.2	0.2 ± 0.1	0.015	0.5 ± 0.3	0.3 ± 0.2	0.005	0.2 ± 0.1	0.1 ± 0.1	0.001
NT-proBNP- pg/ml	1,298.3 ± 205.8	122.5 ± 26.6	<0.001	1,582.4 ± 345.6	131.7 ± 31.7	<0.001	1,321.8 ± 318.2	357.8 ± 86.7	<0.001
D-dimer-ng/ml	2,318.4 ± 333.5	387.3 ± 78.4	<0.001	1891.3 ± 452.8	432.5 ± 52.3	<0.001	1,465.9 ± 388.9	854.0 ± 137.7	0.001

Note: TNI:troponin I; NT-proBNP:N-terminal pro B-type natriuretic peptide.

### Third Outcomes

**FIGURE 2:**
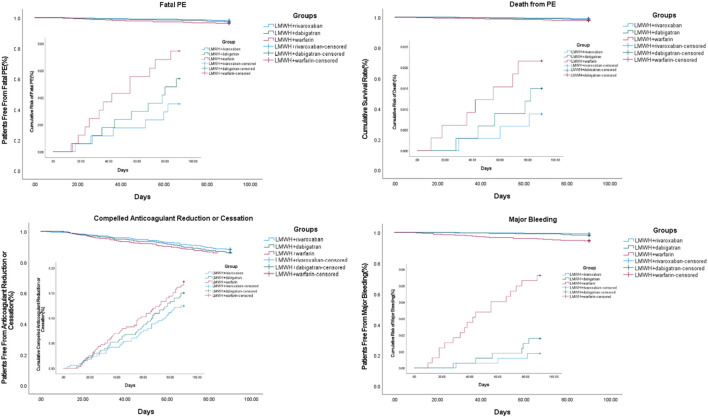
Third and safety outcomes during 3-month follow-up. Note: PE, pulmonary embolism; LMWH, low molecular weight heparin.

The events of progressed fatal PE for which thrombectomy, vena cava filter insertion, or thrombolysis were performed or indicated during the 3-month treatment of acute PE were 6, 9, and 12 in the nadroparin + rivaroxaban (*n* = 343), nadroparin + dabigatran (*n* = 338), and nadroparin + warfarin (*n* = 329) groups, respectively (*p* = 0.404). The death incidences from fatal PE during the 3-month treatment of acute PE were 3, 5, and 7 in the aforementioned three groups, respectively (*p* = 0.310). The compelled anticoagulant reduction or cessation due to adverse events during the 3-month treatment of acute PE was 40, 47, and 52 in the aforementioned three groups, respectively (*p* = 0.297) (Figure 2).

### Safety Outcomes

At the 3 months, the number of patients with major bleeding events was 3, 6, and 18 in the nadroparin + rivaroxaban (*n* = 343), nadroparin + dabigatran (*n* = 338), and nadroparin + warfarin (*n* = 329) groups, respectively. The major bleeding rates were 0.9, 1.8, and 5.5% in the aforementioned three groups, respectively (*p* < 0.001) (Figure 2). 155, 142, and 128 patients of nadroparin + rivaroxaban, nadroparin + dabigatran, and nadroparin + warfarin groups, respectively, returned at 6 months for another follow-up visit. The 6-month follow-up visit showed that VTE recurrence occurred in 32, 35, and 27 patients of nadroparin + rivaroxaban, nadroparin + dabigatran, and nadroparin + warfarin groups, respectively (*p* = 0.143), whereas major bleeding occurred in 4, 8, and 11 patients of the aforementioned three groups (*p* = 0.099). A univariate logistic regression analysis demonstrated that age>65 years, male, active cancer, recent myocardial infarction, history of stroke, arterial hypertension, heparin-induced thrombocytopenia (HIT), and hemoglobin<100 g/L were shared major risk factors for both VTE recurrence and major bleeding. After the next multivariate analysis, all other variables remained correlated with both VTE recurrence and major bleeding except for patients with age>65 years, male sex, arterial hypertension, and hemoglobin<100 g/L (Table 4).

**TABLE 4:** Shared major risk factors for both VTE recurrence and major bleeding.

Variables	Both VTE recurrence and bleeding			
	Univariate OR (95% CI)	*p* Value	Multivariate OR (95% CI)	*p* Value
Age >65 years	1.663 (1.095–2.231)	0.020	1.326 (0.849–1.803)	0.093
Male vs. female	1.782 (1.257–2.307)	0.005	1.238 (0.571–1.905)	0.125
Active cancer: yes vs. no	4.375 (3.278–5.472)	0.001	4.739 (3.373–6.105)	<0.001
Recent myocardial infarction: yes vs. no	5.349 (3.661–7.037)	<0.001	4.936 (2.875–6.997)	<0.001
History of stroke: yes vs. no	3.918 (2.373–5.463)	<0.001	3.580 (2.643–4.517)	<0.001
Arterial hypertension: yes vs. no	1.865 (1.117–2.613)	0.001	1.336 (0.827–3.754)	0.079
HIT: yes vs. no	2.542 (1.773–3.311)	0.001	2.684 (1.848–3.520)	0.001
Hemoglobin<100 g/L	1.774 (0.852–2.696)	0.003	1.317 (0.659–1.975)	0.082

Note:VTE, venous thromboembolism; OR, odds ratio; HIT, heparin-induced thrombocytopenia.

## Discussion

In the current study, the results indicated that the resolution rate of acute PE on imaging after the 3-month initial treatment in the nadroparin + rivaroxaban group was similar to that in the nadroparin + dabigatran group, which were both higher than that in the nadroparin + warfarin group. No difference was found with respect to the prevalence of DVT at the 3-month follow-up visit among the three groups. The NT-proBNP and D-dimer level at the 3-month follow-up visit in the nadroparin + rivaroxaban group was similar to those in the nadroparin + dabigatran group, which were both lower than those in the nadroparin + warfarin group, respectively. The level change of NT-proBNP and D-dimer from the baseline through the 3-month follow-up visit was similar to those in the nadroparin + dabigatran group, which were both more than those in the nadroparin + warfarin group, respectively. The troponin I level and prevalence of PH-likely patients at the 3-month follow-up visit were similar among the three groups, respectively. The level change in troponin I from the baseline through the 3-month follow-up visit was similar among the three groups. During the 3-month treatment of acute PE, the events of progressed fatal PE, death from fatal PE, and compelled anticoagulant reduction or cessation due to adverse events were similar among the three groups, respectively. The major bleeding rates were similar between the nadroparin + rivaroxaban and nadroparin + dabigatran groups, both being lower than those of the nadroparin + warfarin group.

To our best knowledge, this is the first study that assessed the efficacy and safety of sequential subcutaneous LMWH followed by oral rivaroxaban therapy in the treatment of patients with acute pulmonary embolism. The comparable studies to the current one are lacking, despite some relevant studies. In an observational study, 99 patients with active cancer and an established VTE receiving sequential treatment of dalteparin [9 days (5–20 days)] and rivaroxaban [2.8 months (1–6 months)] were retrospectively reviewed. One (1.0%) patient developed pulmonary embolism, and seven (7.1%) patients suffered recurrent VTE, without major bleeding being observed during the 6-month follow-up period. Sequential rivaroxaban after the dalteparin regimen could effectively reduce the risk of VTE recurrence with well tolerance (Chen et al., 2021). In another retrospective study, no symptomatic recurrence or major bleeding events were encountered after the 6-month follow-up among 49 patients with confirmed VTE receiving an initial therapy with 1–18 days of parenteral enoxaparin (1 mg/kg twice daily) followed by oral rivaroxaban 20 mg every day. It was effective and safe to treat patients with established VTE by using a regimen of enoxaparin followed by a dose of 20 mg of rivaroxaban (Wolosker et al., 2016). In an open-label, single-arm, multicenter study, no occurrence of VTE or bleeding events was encountered among 56 patients who had undergone elective unilateral primary total hip replacement (THR) or total knee replacement (TKR) and subcutaneous enoxaparin (40 mg once daily or 30 mg twice daily) followed by oral rivaroxaban (10 mg once daily, 35 days for THR and 14 days for TKR) for postoperative thromboprophylaxis. Sequential LMWH and rivaroxaban regimen provided an effective and well-tolerated strategy for thromboprophylaxis in patients undergoing THR/TKR surgery (Mills et al., 2012).

The efficacy and safety of rivaroxaban monotherapy have been extensively validated in previous studies. In the EINSTEIN-PE trial, the symptomatic recurrent VTE events were 50 (2.1%) in the rivaroxaban (15 mg twice daily for 3 weeks, followed by 20 mg once daily) group (*N* = 2,419) and 44 (1.8%) in the standard-therapy (enoxaparin followed by a dose-adjusted VKA) group (*N* = 2,413), during a 12-month follow-up. The principal safety outcome occurred in 249 (10.3%) patients of the rivaroxaban group and 274 (11.4%) ones of the standard therapy group (*p* = 0.23). Rivaroxaban monotherapy was noninferior to standard therapy which was defined as sequential LMWH and VKA for the initial and long-term treatment of acute PE (EINSTEIN–PE Investigators et al., 2012). In a propensity-matched cohort study, the incidence of recurrent VTE after the 6-month follow-up was 9.9 incidents per 100 person-years with rivaroxaban (*N* = 1734) and 13.1 incidents per 100 person-years with warfarin (*N* = 2,945) [hazard ratio (HR) 0.74 (95% CI 0.56–0.96)]. The incidence of major bleeding events was 2.4 per 100 person-years at 6 months with rivaroxaban versus 2.0 with warfarin (HR 1.19, 95% CI 0.66–2.13). Rivaroxaban was associated with reduced risk of recurrent VTE compared with the standard regimen for patients with unprovoked VTE, without safety concerns (Larsen et al., 2017). In addition, several studies demonstrated that rivaroxaban was similar to dabigatran for the treatment of acute VTE, in terms of VTE recurrence, VTE-related death, and all-cause mortality, except that rivaroxaban seemed to result in a higher bleeding risk than that in dabigatran (Rollins et al., 2014; Cohen et al., 2015; Mantha and Ansell, 2015; Jugrin et al., 2016). The efficacy and safety of sequential therapy of body-weight adjusted nadroparin followed by oral rivaroxaban at a dose of 20 mg once daily in the current study are basically consistent with those of rivaroxaban monotherapy in previous studies.

The current study suffers from some limitations. First of all, it was a retrospective study. A prospective study is warranted in the future. Second, since rivaroxaban monotherapy (15 mg twice daily for 21 days followed by 20 mg once daily) is not a popular regimen in the participating hospitals even in the whole country because parenteral drug-delivery is preferred over oral one during hospitalization due to the traditional concept of Chinese patients, there is no way to learn the results of head-to-head comparison between rivaroxaban monotherapy and sequential LMWH and rivaroxaban regimen. Third, since patients receiving dabigatran at a dose of 110 mg twice daily were not included in the current study, the results may not be applicable to this patient population. Fourth, most patients in the nadroparin + warfarin group were those who were treated between 2010 and 2015 since DOACs were still not extensively used in China then, whereas most patients in the other two groups were those who were treated between 2016 and 2020. This might yield heterogeneity in the study population. Nevertheless, the diagnostic, therapeutic, and follow-up procedures were consistent between patients from 2010 through 2015 and those from 2016 through 2020. A subgroup analysis demonstrated that the major demographics, such as age and body weight, were similar between patients from 2010 through 2015 and those from 2016 through 2020. Fifth, since the LMWH used in the present study was nadroparin instead of enoxaparin or dalteparin, which are used as initial treatment of acute PE in most other published studies, there is no way to know what the present results would be if enoxaparin or dalteparin had been substituted for nadroparin. The last but not the least since the present study did not include patients with COVID-19, the results may not be applicable for the patient population that is prone to suffer from acute PE.

In conclusion, the current study suggests that compared with sequential nadroparin plus warfarin and nadroparin plus dabigatran, the regimen of sequential subcutaneous nadroparin for a week followed by oral rivaroxaban at a dose of 20 mg once daily for 3 months is effective and safe in the initial treatment of patients with acute pulmonary embolism. This finding may provide clinicians with more options with respect to the initial treatment of patients with acute pulmonary embolism.

## Data Availability

The original contributions presented in the study are included in the article/Supplementary Material; further inquiries can be directed to the corresponding authors.
